# *Geobacillus zalihae *sp. nov., a thermophilic lipolytic bacterium isolated from palm oil mill effluent in Malaysia

**DOI:** 10.1186/1471-2180-7-77

**Published:** 2007-08-10

**Authors:** Raja Noor Zaliha Raja Abd Rahman, Thean Chor Leow, Abu Bakar Salleh, Mahiran Basri

**Affiliations:** 1 Enzyme and Microbial Technology Research Group, Faculty of Biotechnology and Biomolecular Sciences, Universiti Putra Malaysia, 43400 UPM Serdang, Selangor, Malaysia; 2 Enzyme and Microbial Technology Research Group, Faculty of Science, Universiti Putra Malaysia, 43400 UPM Serdang, Selangor, Malaysia

## Abstract

**Background:**

Thermophilic *Bacillus *strains of phylogenetic *Bacillus *rRNA group 5 were described as a new genus *Geobacillus*. Their geographical distribution included oilfields, hay compost, hydrothermal vent or soils. The members from the genus *Geobacillus *have a growth temperatures ranging from 35 to 78°C and contained iso-branched saturated fatty acids (iso-15:0, iso-16:0 and iso-17:0) as the major fatty acids. The members of *Geobacillus *have similarity in their 16S rRNA gene sequences (96.5–99.2%). Thermophiles harboring intrinsically stable enzymes are suitable for industrial applications. The quest for intrinsically thermostable lipases from thermophiles is a prominent task due to the laborious processes via genetic modification.

**Results:**

Twenty-nine putative lipase producers were screened and isolated from palm oil mill effluent in Malaysia. Of these, isolate T1^T ^was chosen for further study as relatively higher lipase activity was detected quantitatively. The crude T1 lipase showed high optimum temperature of 70°C and was also stable up to 60°C without significant loss of crude enzyme activity. Strain T1^T ^was a Gram-positive, rod-shaped, endospore forming bacterium. On the basic of 16S rDNA analysis, strain T1^T ^was shown to belong to the *Bacillus *rRNA group 5 related to *Geobacillus thermoleovorans *(DSM 5366^T^) and *Geobacillus kaustophilus *(DSM 7263^T^). Chemotaxonomic data of cellular fatty acids supported the affiliation of strain T1^T ^to the genus *Geobacillus*. The results of physiological and biochemical tests, DNA/DNA hybridization, RiboPrint analysis, the length of lipase gene and protein pattern allowed genotypic and phenotypic differentiation of strain T1^T ^from its validly published closest phylogenetic neighbors. Strain T1^T ^therefore represents a novel species, for which the name *Geobacillus zalihae *sp. nov. is proposed, with the type strain T1^T ^(=DSM 18318^T^; NBRC 101842^T^).

**Conclusion:**

Strain T1^T ^was able to secrete extracellular thermostable lipase into culture medium. The strain T1^T ^was identified as *Geobacillus zalihae *T1^T ^as it differs from its type strains *Geobacillus kaustophilus *(DSM 7263^T^) and *Geobacillus thermoleovorans *(DSM 5366^T^) on some physiological studies, cellular fatty acids composition, RiboPrint analysis, length of lipase gene and protein profile.

## Background

The *Bacillus *rRNA group 5 which comprised thermophilic *Bacillus *strains was transferred into new genus *Geobacillus *which represented a phenotypically and phylogenetically coherent group of thermophilic bacilli with high levels of 16S rRNA sequence similarity (96.5–99.2%) [1]. The members of this genus are widespread in various thermophilic and mesophilic geographic areas on the earth such as oilfields, hay compost, hydrothermal vent or soils [1-5]. At present, the members of this genus included *Geobacillus stearothermophilus*, *Geobacillus thermocatenulatus*, *Geobacillus thermoleovorans*, *Geobacillus kaustophilus, Geobacillus thermoglucosidasius*, *Geobacillus thermodenitrificans*, *Geobacillus subterraneus*, *Geobacillus uzenensis*, *Geobacillus caldoxylosilyticus*, *Geobacillus toebii*, *Geobacillus vulcani*, *Geobacillus lituanicus*, *Geobacillus tepidamans*, *Geobacillus gargensis*, *Geobacillus jurassicus*, *Geobacillus caldoproteolyticus*, *Geobacillus pallidus *and *Geobacillus debilis *with growth temperatures ranging from 35 to 78°C [1,4-19].

Recently, microorganisms such as *Bacillus *sp. RSJ-1 [20], *Bacillus thermoleovorans *ID-1 [21], *Bacillus *sp. THL027 [22], *Bacillus *sp. strain A30-1 [23], *Bacillus *sp. strain 398 [24], *Bacillus thermocatenulatus *[25], *Bacillus *spp. [26-30] were reported as thermostable lipase producers. As thermophilic bacterial strains have an optimum growth temperature of 65–70°C, lipases isolated from such strains are good candidates for lipid modifications [31].

The stability of biocatalysts is an important criterion when dealing with bioprocesses at high temperature for sustainable operation. Enzyme stability is dictated by its three-dimensional configuration, which in turn is determined by genetic and environmental factors [32]. Therefore, thermophiles are promising sources of heat-stable enzymes. In addition to higher thermostability, proteins from thermophiles often showed higher stability toward organic solvents and higher activity at elevated temperature [33]. In addition, genetic engineering in altering the stability of enzymes is a difficult task and laborious processes. Therefore, efforts have been focused on the screening of microorganisms harboring intrinsically stable biocatalysts. Putative lipase producers were screened quantitatively across various basal media to check for lipase production and the stability of lipase. The highest lipase producing strain have been identified and characterized intensively. Screening and isolation of heat-stable lipase producers are important to fulfill industrial requirements for the desired characteristics. Identification of industrially important enzyme producer is conducted to determine its phylogenetic position in systematic microbiology.

## Results and discussion

### Screening and isolation of thermophilic lipolytic bacteria

Rubbish dump sites and palm oil mill effluent are potential sites containing thermophilic lipolytic bacteria because these sites served as sewage discharge areas for household waste and palm oil factory. The oily environment may provide a good environment for lipolytic microorganisms to flourish. Samples were collected from the rubbish dump site and palm oil mill effluent at different treatment areas. Samples were enriched with enrichment medium (EM-1) containing olive oil as the sole carbon source at 60°C for 2 days under shaking condition to promote the growth of thermophilic lipolytic bacteria. Twenty nine putative lipase producers gave positive results on triolein agar plate by forming an intense blue color around the colonies (Table 1). Of these, 23 and 6 isolates were isolated from rubbish dump site and palm oil mill, respectively. Further confirmation test was performed quantitatively on the 29 putative lipase producers that gave positive results on triolein agar plates. Various basal media M1, M3, TYEM and BM-1 were tested for lipase production during the selection. Low lipase activities ranging from 0 ~ 0.044 U/ml were detected colorimetrically in culture supernatant, except for isolate T1^T^, which showed high lipase activity (0.150 U/ml) after 24 h incubation (Table 1).

**Table 1:** Qualitative and quantitative assay of different isolates.

Isolates	Triolein agar (at 60°C)	Lipase activity (U/ml)			
		
		M1	TYEM	M3	BM1
*Rubbish dump site:*					
AP1	+	-	-	-	-
4	+	0.040	0.006	0.004	-
TL1	+	-	-	0.030	-
S44	+	0.025	0.038	-	-
2	+	-	0.025	-	-
H	+	0.031	-	-	-
X	+	-	0.009	-	-
O	+	0.018	-	-	-
P	+	-	-	-	-
N	+	0.004	-	-	-
AP3	+	-	-	0.044	-
S1	+	0.003	0.029	-	-
J	+	-	0.014	-	-
AP2	+	-	0.014	0.022	-
SS6	+	-	0.004	-	-
SS5	+	-	-	-	-
F	+	-	-	-	-
Q	+	0.011	0.040	-	-
Y	+	0.012	-	-	-
3	+	-	-	-	-
C	+	-	-	-	-
D	+	0.006	-	-	-
M	+	-	0.010	-	-
*Palm oil mill effluent:*					
W5	+	-	-	-	0.005
W6	+	-	-	-	0.009
W7	+	-	-	-	0.009
T1^T^	+	-	-	-	0.150
T2	+	-	-	-	0.041
T3	+	-	-	-	0.011

Note: (+), indicates formation of intense blue colour on triolein agar plate

**Figure 1:**
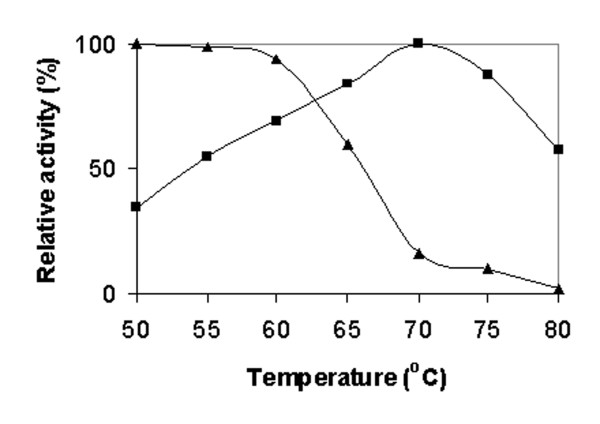
Lipase activity (■) and stability (▲) of crude T1 lipase at various temperature. Crude T1 lipase was assayed at various temperatures ranging from 50 to 80°C with olive oil emulsion (1:1, v/v) as substrate (pH 7.0). For the lipase stability test, crude T1 lipase was assayed after heat treatment at various temperatures for 30 min.

On the basis of relatively higher lipase activity detected for isolate T1^T^, the effect of temperature on the activity and stability of crude T1 lipase was further investigated. The effect of temperature on the lipase activity and stability was examined from 40 to 80°C. As shown in Fig. 1, the crude enzyme from isolate T1^T ^manifested its maximal activity at 70°C with olive oil as substrate. Crude T1 lipase was fairly stable up to 60°C for 30 min and gradually decreased upon prolonged temperature treatment. This is due to T1 lipase tended to lose its native conformation as a result of breaking of the intrinsic interaction above its stable range. This is a discrepancy between temperature activity (70°C) and stability (60°C), as the enzyme is tend to be protected by heat denaturation in the presence of olive oil. In addition, the enzyme thermostability is greatly influenced by the presence of water, because denaturation is linked to its conformational mobility in aqueous mixture [34]. The crude enzyme of isolate T1^T ^was fairly active at higher temperature as compared to other thermostable lipases from *Bacillus *spp. [26,30,33] which exhibited maximal activity at 60°C. High temperature activity and stability of enzyme offer great potentials in industrial applications, and hence attempts have been made to identify isolate T1^T^.

### Identification of isolate T1^T^

During the characterization of organism isolated from palm oil mill effluent, strain T1^T ^was recovered on nutrient broth at 60°C. The growth condition for strain T1^T ^was 50–70°C and between pH 5 and 9 with the optimum growth temperature and pH of 65°C and pH 6.5, respectively, in nutrient broth. These met the criteria of thermophilic bacteria, which grew at temperatures above 50°C [35]. To verify the systematic position of this bacterium, a study of morphological and physiological characteristics, 16S rRNA analysis, cellular fatty acids analysis, DNA composition, DNA/DNA hybridization, RiboPrint analysis, lipase gene analysis and protein profile were undertaken.

**Figure 2:**
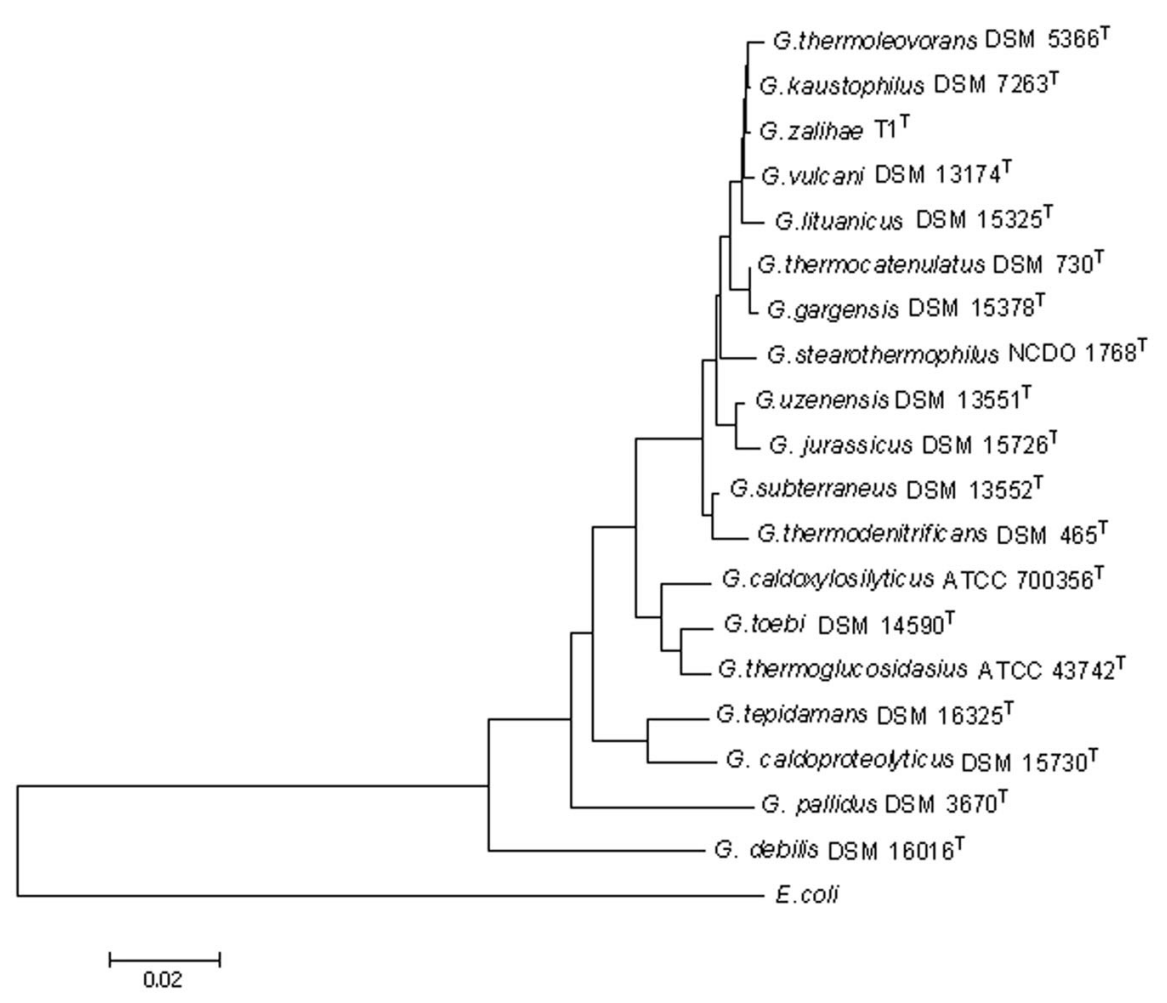
Phylogenetic position of *Geobacillus zalihae *T1^T ^with other validly described species of the genus *Geobacillus*. The members of genus *Geobacillus *used include *G. thermoleovorans *(DSM 5366^T^); *G. kaustophilus *(DSM 7263^T^); *G. vulcani *(DSM 13174^T^); *G. lituanicus *(DSM 15325^T^); *G. thermocatenulatus *(DSM 730^T^); *G. gargensis *(DSM 15378^T^); *G. stearothermophilus *(NCDO 1768^T^); *G. uzenensis *(DSM 13551^T^); *G. jurassicus *(DSM 15726^T^); *G. subterraneus *(DSM 13553^T^); *G. thermodenitrificans *(DSM 465^T^); *G. caldoxylosilyticus *(ATCC 700356^T^); *G. toebi *(DSM 14590^T^); *G. thermoglucosidasius *(ATCC 43742^T^) *G. tepidamans *(DSM 16325^T^); *G. caldoproteolyticus *(DSM15730^T^); *G. pallidus *(DSM3670^T^); *G. debilis *(DSM16016^T^). *Escherichia coli *were used as an out-group. Phylogenetic tree was inferred by using the neighbour-joining methods. The software package MEGA 3.1 was used for analysis.

The cellular morphology of isolate T1^T ^is rod-shaped, 0.8–1.0 μm width and 2.5–6.0 μm length, gram positive bacteria. The terminal spore is oval/cylindrical in shape and swollen the sporangium. The DNA base composition of strain T1^T ^is around 52.6% mol G + C. The partial sequencing of the 16S rDNA shows 99.5% similarity to validly described *Geobacillus kaustophilus *(DSM 7263^T^) and *Geobacillus thermoleovorans *(DSM 5366^T^). The 16S rRNA sequence of strain T1^T ^is a continuous stretch of 1519 bp (AY166603). Construction of phylogenetic trees using the neighbour-joining method in determining the evolutionary relationship among a group of validly described closely related species is indicated in Fig. 2. A comparison of biochemical, morphological and physiological properties of strain T1^T ^with its closest phylogenetic neighbors is presented in Table 2. Strain T1^T ^can be distinguished from *Geobacillus thermoleovorans *(DSM 5366^T^) phenotypically by oxidase test, arabinose, mannitol, inositol, lactose and casein hydrolysis. However, strain T1^T ^differs from *Geobacillus kaustophilus *(DSM 7263^T^) by lysozyme test, arabinose, mannitol, ribose, adonitol, lactose, gelatin and casein tests. Since the sequencing result and physiological data did not allow strain T1^T ^to be identified with one of the above mentioned species, further analysis need to be carried out to verify its phylogenetic position.

**Table 2:** The differentiating characteristics of the thermophilic strain T1^T ^and its type strains.

Characteristics	1	2	3
Cell width (μm)	0.8–1.0	≥ 0.9	1.5
Cell length (μm)	2.5–6.0	≥ 3	3.5
Spores oval/cylindrical	O/C	O/C	O
Spores position	T	T	T
Oxidase	-	-	+
Growth in:			
NaCl 2%	+	+	ND
5%	-	+	ND
Lysozyme broth	-	+	ND
Production of acid from:			
L-arabinose	+	-	-
D-xylose	+	+	v
D-mannitol	-	+	+
M-inositol	+	+	-
D-ribose	+	-	+
D-cellobiose	w	+	+
D-galactose	+	v	+
Adonitol	-	+	v
D-lactose	+	-	-
Hydrolysis of:			
Gelatin	-	+	-
Casein	-	+	+
Nitrate reduction	+	+	ND
pH range	5.0–9.0	6.0–8.0	6.2–7.5
Temperature (°C)	50–70	37–68	45–70
DNA G+C content (mol %)	52.6	51–55	52–58

Taxa 1, *G. zalihae *T1^T^; 2, *G. Kaustophilus *(DSM 7263^T^); 3, *G. thermoleovorans *(DSM 5366^T^). Characteristics are scored as: +, positive; -, negative; w, weak; v, variable within the group; ND, not determined. Data were obtained from the present study (*G. zalihae *strain T1^T^), Nazina *et al*. [16], Sunna *et al*. [3], Kuisiene *et al*. [14] and Markossian *et al*. [54] (*G. thermoleovorans *DSM 5366^T ^and *G. Kaustophilus *DSM 7263^T^). All strains were negative for anaerobic growth, growth at 30°C, VP reaction, indole production and acid from L-rhamnose, sorbitol. All strains were positive for catalase, hydrolysis of starch, use of citrate and acid from D-fructose and D-glucose.

Result of chemotaxonomic analyses is given in the species description. The fatty acid profile of strain T1^T ^is typical for the *Bacillus *rRNA-group 5 (thermophilic *Bacillus *strains). The major content of cellular fatty acids of strain T1^T ^is iso-fatty acids. Among them, iso-branched pentadecanoic acid (iso-C15), hexadecanoic acid (iso-C16) and heptadecanoic acid (iso-C17) making up 78.33% of the total fatty acids for strain T1^T^. Strain T1^T ^can be differentiated from *Geobacillus thermoleovorans *DSM 5366^T ^based on percent composition of iso-fatty acids (iso-C15, iso-C16 and iso-C17 making up 62.1%). There was only 6.14% of iso-C16 for strain T1^T ^but 21% for *Geobacillus thermoleovorans *DSM 5366^T ^(Table 3).

**Table 3:** Cellular fatty acids composition of *Geobacillus zalihae *T1^T ^and its phylogenetical neighbors.

Fatty acid	*Geobacillus zalihae *T1^T^	*Geobacillus thermoleovorans *(DSM 5366^T^)
10:0	-	2.7
14:0 ISO	-	1.0
14:0	7.22	1.4
15:0 ISO	32.42	22.6
15:0 ANTEISO	1.01	1.3
15:0	0.82	2.1
16:0 ISO	6.14	21.0
16:0	4.98	11.2
17:0 ISO	39.77	18.5
17:0 ANTEISO	4.97	4.6
17:0	0.53	1.3
18:1 ISO H	0.38	-
18:1 ISO	0.36	0.9
18:0	0.47	3.4
18:1	-	1.2
19:0 ISO	0.91	-
Unsaturated C16	-	6.6
Other	-	0.2

Note: Data were obtained from the present study (*G. zalihae *strain T1^T^), Nazina *et al*. [1] (*G. thermoleovorans *DSM 5366^T^).

Although FAME is not always a reliable tool for preliminary identification of bacilli, it could be used in combination with other identification methods [36]. DNA/DNA hybridization experiments were performed in DSMZ (Germany) with strain T1^T ^and its type strains of closest phylogenetical neighbors. The genomic DNA/DNA relatedness between strain T1^T ^and its type strains *Geobacillus kaustophilus *(DSM7263^T^) *Geobacillus thermoleovorans *(DSM 5366^T^) were 73.6% and 68.2%, respectively. Whereas, the type strains *Geobacillus kaustophilus *(DSM 7263^T^) and *Geobacillus thermoleovorans *(DSM 5366^T^) showed a DNA/DNA similarity of 71.8%. The DNA/DNA reassociation values were below the threshold value of 70% DNA/DNA similarity for definition of species [37] between strain T1^T ^and *Geobacillus thermoleovorans *DSM 5366^T ^but above the threshold between strain T1^T ^and *Geobacillus kaustophilus *DSM 7263^T^. Neither the species *Geobacillus kaustophilus *DSM 7263^T ^and *Geobacillus thermoleovorans *DSM 5366^T ^can be differentiated from one another nor strain T1^T ^can be differentiated at the species level from its closest phylogenetic neighbors by DNA/DNA hybridization. However, DNA/DNA hybridization tests between *Geobacillus kaustophilus *DSM 7263^T ^and *Geobacillus thermoleovorans *DSM 5366^T ^were 84% and 54% as reported by Sunna *et al*. [3] and Nazina *et al*. [13]. This disagreement may be due to the absence of adequate hybridization controls in the experiments. Therefore, further tests need to be carried out to accurately place the strain T1^T ^phylogenetically.

The RiboPrint analysis was carried out for the decision on the affiliation of strain T1^T^. However, the RiboPrint pattern of strain T1^T ^was not identified by the Dupont identification library to give rise to the identification at the species level (>0.85). Its RiboPrint pattern showed the highest similarity to *Geobacillus kaustophilus *DSM 7263^T ^(0.69). The similarity to the pattern of *Geobacillus thermoleovorans *DSM 5366^T ^was somewhat lower (0.57). The patterns between type strains *Geobacillus kaustophilus *DSM 7263^T ^and *Geobacillus thermoleovorans *DSM 5366^T ^show a binary similarity of 0.64.

**Figure 3:**
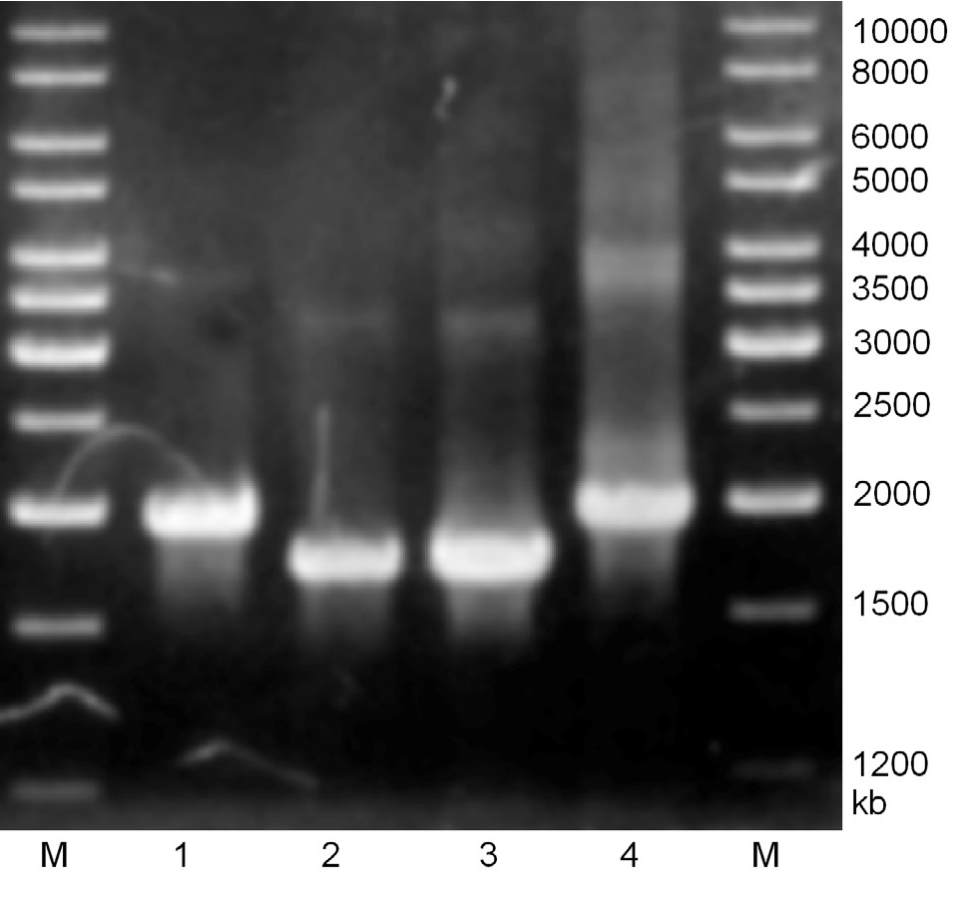
Amplification of thermostable lipase gene. M: Marker; 1: Control recombinant plasmid harboring thermostable lipase gene; 2: DSM 5366^T^; 3: DSM 7263^T^; 4: T1^T^.

**Figure 4:**
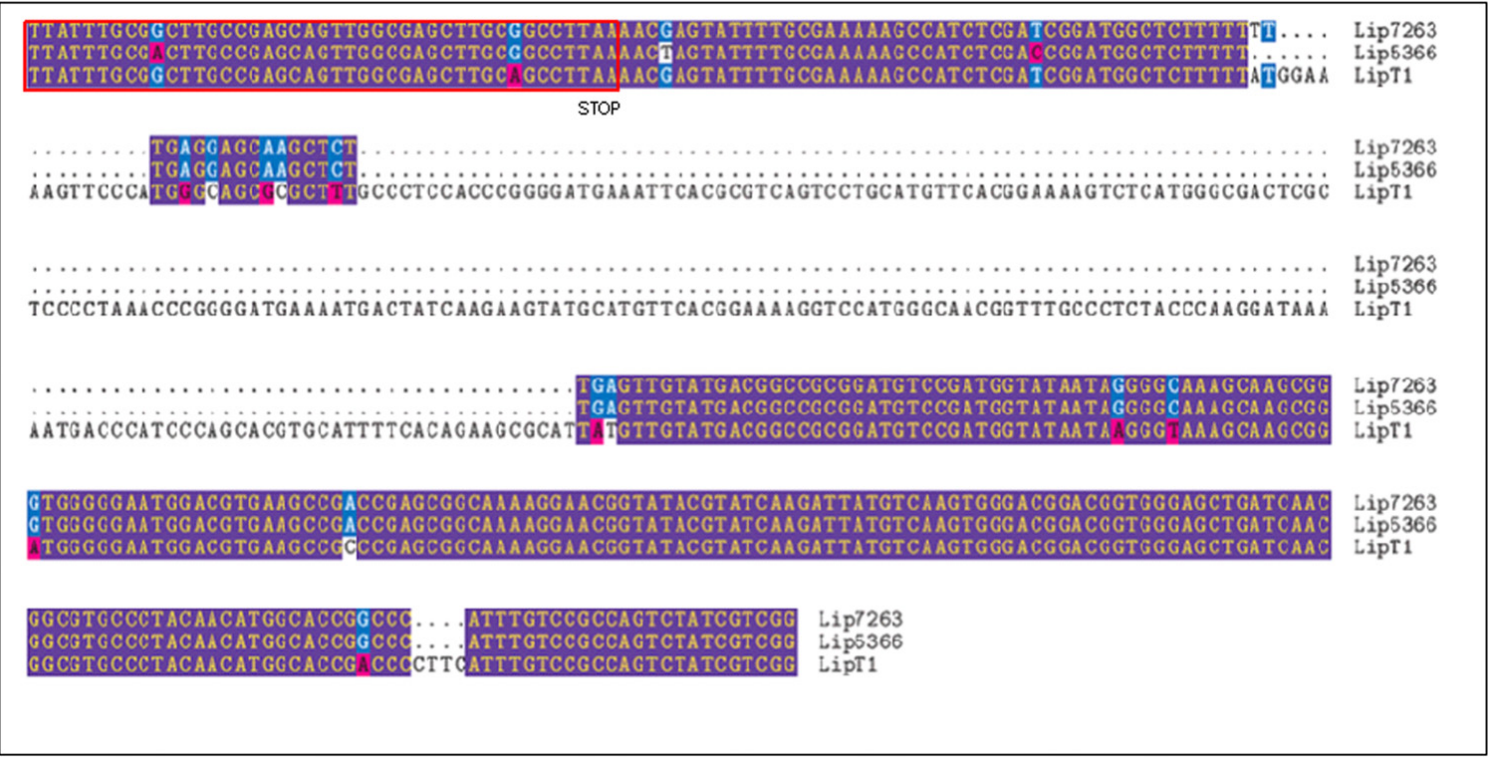
Downstream sequence alignment of lipase genes derived from *Geobacillus zalihae *T1^T ^and its phylogenetic neighbors. The alignment was generated using Lip7263 (*G. Kaustophilus *DSM 7263^T^), Lip5366 (*G. thermoleovorans *DSM 5366^T^), and LipT1 (*G. zalihae *T1^T^). The C-terminal of the open reading frame coding sequences is bracket in red, and the stop codon was labeled as STOP.

**Figure 5:**
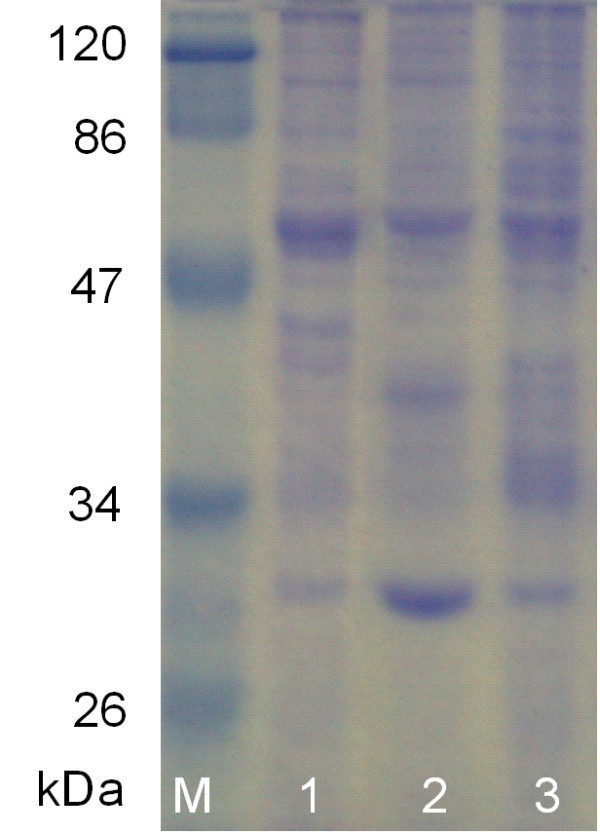
Protein pattern of *Geobacillus zalihae *T1^T ^and its phylogenetic neighbors. M: Marker; 1: DSM 5366^T^; 2: DSM 7263^T^; 3: T1^T^.

Further analysis was also carried out by amplifying full-length thermostable lipase gene using primers as described in materials and methods [38]. Fig. 3 showed amplified full-length lipase genes of strain T1^T ^and its type strains. The amplified lipase gene of strain T1^T ^was around 2 kb but 1.8 kb for its type strains *Geobacillus kaustophilus *DSM 7263^T ^and *Geobacillus thermoleovorans *DSM 5366^T^. A stretch of about 220 bp insertion could be seen at downstream of the open reading frame of thermostable lipase gene (Fig. 4). In addition, the intracellular protein profiles were determined by SDS-PAGE. Samples (30 μg) were separated on 12% SDS-polyacrylamide gel and stained using Coomassie blue. Strain T1^T ^showed obvious different protein profile as compared to its type strains at region between 34 to 47 kDa (Fig. 5).

### Description of *Geobacillus zalihae *sp. nov

*Geobacillus zalihae *(za.li'ha.e. N.L. gen. n. zalihae of Zaliha). The novel species is isolated from palm oil mill effluent in Selangor, Malaysia, with the type strain T1^T ^(DSM 18318^T^; NBRC 101842^T^). Cells are rod-shaped, 0.8–1.0 width and 2.5–6.0 length, gram positive bacteria. The terminal spores are oval/cylindrical and swollen the sporangium. Growth occurs at 50–70°C with an optimum temperature of 65°C. Growth at 65°C occurs between pH 5 and 9 with maximal growth at pH 6.5. The DNA base composition of strain T1^T ^was around 52.6% mol G + C. The iso-fatty acids were in major amount according to cellular fatty acid profile in which iso-C15 (32.42%), and iso-C17 (39.77%) were in abundant (77.19%). Growth is aerobic and tolerant up to 2% NaCl. It can not perform anaerobic growth. It shows positive in catalase test but not oxidase test. It is able to hydrolyze starch but not gelatin and casein. Acids are produced from L-arabinose, D-lactose but not D-mannitol.

## Conclusion

As a consequence, the strain T1^T ^merits recognition as a member of a novel species through morphological and physiological studies, cellular fatty acids composition, DNA composition, DNA/DNA hybridization, RiboPrint analysis. Sizes of full-length lipase genes and protein profiles were additional evidences. The *Geobacillus zalihae *strain T1^T ^was deposited in DSMZ (DSM 18318^T^) and NITE (NBRC 101842^T^).

## Methods

### Bacterial isolation

Numerous enrichment cultures that were derived from samples of rubbish dump site and palm oil mill effluent were screened for their ability to degrade olive oil. The strain described here was isolated from a sample from palm oil mill effluent in Semenyih, Malaysia. Samples (5%) were inoculated into enrichment medium (EM1) with olive oil as the sole carbon source and incubated at 60°C under shaking condition (150 rpm) for 2 days at an initial pH 7.0. The composition of EM1 is as follows: olive oil 2%; NaCl 0.2%; MgSO_4_.7H_2_O 0.04%; MgCl_2_.6H_2_O 0.07%; CaCl_2_.2H_2_O 0.05%; KH_2_PO4 0.03%; K_2_HPO_4 _0.03%; (NH_4_)_2_SO_4 _0.05% [21], to which 0.01% of trace elements solution containing 0.026% B, 0.05% Cu, 0.05% Mn, 0.006% Mo and 0.07% Zn were added [27]. The enriched cultures were further screened by using triolein agar plate. Triolein agar comprising of triolein (0.25%), bacteriological agar (1%), nutrient broth (0.8%) and Victoria Blue (0.01%) was adjusted to pH 7.0. The medium was homogenized for 5 minutes before sterilization. The sterilized triolein agar was poured into petri dishes. Isolates that showed positive results on the triolein agar were then tested for their lipase production in basal media.

### Selection of thermostable lipase producer

Lipase production was determined aerobically at 60°C in 500 ml blue cap bottle containing 100 ml basal medium. The composition of basal mineral media used in this study was (g/L): BM1 (NaNO_3_: 7; K_2_HPO_4_: 2; KH_2_PO_4_: 1; KCl: 0.1; MgSO_4_.7H_2_O: 0.5; CaCl_2_: 0.01; FeSO_4_.7H_2_O: 0.012; yeast extract: 1 in which 0.01% trace elements and 2% olive oil were supplemented) [39]; M1 (peptone: 3; yeast extract: 1; NaCl: 0.5 in which 1% olive oil was supplemented) [30]; M3 (nutrient broth: 0.325; gum Arabic: 1; CaCl_2_.2H_2_O: 0.05; Tween.80: 1 in which 1% olive oil was supplemented) [40] and TYEM (tryptone: 6; yeast extract: 2; CaCl_2_.2H_2_O: 0.2; MgSO_4_.7H_2_O: 0.1; FeCl_3_.6H_2_O: 0.4 in which 1.5% olive oil was supplemented) [21]. The pH was adjusted to 7.0 and the medium was sterilized for 15 min at 121°C. Bacterial inoculum (3 ml) was then inoculated into 100 ml basal medium and incubated by shaking at 150 rpm, 60°C. The cell free supernatant was obtained by centrifugation at 10, 000 rpm, 4°C for 10 min prior to lipase assay.

### Lipase assay

The lipase activity was assayed by colorimetry [41]. Culture filtrate (1 ml) was shaken with 2.5 ml of olive oil (70% oleate residues) emulsion (1:1, v/v) and 20 μl of 0.02 M CaCl_2 _in a water bath shaker at an agitation rate of 200 rpm. The emulsion was prepared by mixing together an equal volume of olive oil (Bertoli, Italy) and 50 mM phosphate buffer (pH 7.0) with a magnetic stirrer for 10 min. The reaction mixture was shaken for 30 min at 50°C. The enzyme reaction in the emulsion system was stopped by adding 6 M HCl (1 ml) and isooctane (5 ml), followed by mixing using a vortex mixer for 30 s. The upper isooctane layer (4 ml) containing the fatty acid was transferred to a test tube for analysis. Copper reagent (1 ml) was added and again mixed with a vortex mixer for 30 s. The reagent was prepared by adjusting the solution of 5% (w/v) copper (II) acetate-1-hydrate to pH 6.1 with pyridine. The absorbance of the upper layer was read at 715 nm. Lipase activity was measured by measuring the amount of free fatty acids released based on the standard curve of free fatty acid. One unit of lipase activity was defined as the amount of enzyme releasing 1 μmole of fatty acid per minute.

### Characterization of crude lipase

The effect of temperature of the crude lipase was evaluated by assaying at temperatures ranging from 50 to 80°C. Crude enzyme (1 ml) was shaken with 2.5 ml of olive oil (70% oleate residues) emulsion (1:1, v/v) and 20 μl of 0.02 M CaCl_2 _in a water bath shaker at an agitation rate of 200 rpm. The lipase activity was measured colorimetrically. Assessment of the thermostability of crude lipase was performed by measuring the residual activity after 30 min pre-incubation at various temperatures ranging from 50 to 80°C. The treated enzyme was immediately put in ice-bath for 10 min before measuring the residual activity at 50°C for 30 min.

### Identification of strain T1^T^

A study of morphological and 16S rRNA analysis was conducted in UPM. Its physiological characteristics, cellular fatty acids analysis, DNA composition, DNA/DNA hybridization and RiboPrint analysis was undertaken in Deutsche Sammlung Von Mikroorganismen (DSMZ), Germany.

#### Morphological and physiological study

For the morphological study, pure bacterial strain was streaked on nutrient agar plate and incubated for 24 h at 60°C prior to gram staining. It was then observed under a light microscope. Morphological and physiological characteristics were further determined in DSMZ (Germany). The physiological characteristics study included catalase and oxidase test, anaerobic growth, Voges-Proskauer test, growth at 30, 40 and 70°C, growth in medium at pH 5.7, 2% and 5% NaCl, lysozyme broth, fermentation of D-glucose, L-arabinose, D-xylose, D-mannitol, D-fructose, M-inosit, D-ribose, D-cellobiose, L-rhamnose, sorbitol, D-galactose, adonit, D-lactose, hydrolysis of starch, gelatin, casein and Tween 80, decomposition of tyrosine, use of citrate and propionate, nitrate reduction, indol production, phenylalanine deaminase and arginine dihydrolase test.

#### Cellular fatty acids analysis

Fatty acids were extracted and analysed following the instructions of Sherlock microbial identification system. The culture of *Bacillus thermocatenulatus *was used as control during cellular fatty acids analysis.

#### 16S rDNA analysis

The 16S rDNA was amplified by PCR using two universal primers: 16S-F (5'-GAG TTT GAT CCT GGC TCA G-3') and 16S-R (5'-CGG CTA CCT TGT TAC GAC TT-3'). The PCR product was purified using QIAquick gel extraction kit (Qiagen, Germany). The purified PCR product was cloned into TOPO TA PCR 2.1 cloning vector (Invitrogen, USA). The recombinant plasmid was extracted with QIAprep plasmid extraction kit (Qiagen, Germany) and was then sequenced using an ABI PRISM 377 DNA sequencer (Applied Biosystems, USA) as recommended by the manufacturers. The 16S rDNA sequence of *Geobacillus zalihae *T1^T ^was analyzed using software package MEGA 3.1 [42].

#### DNA base composition

The chromosomal DNA was isolated and purified according to the procedure of Cashion *et al*. [43]. The G + C contents were determined by using chromatography conditions adopted from Tamaoka and Komagata [44]. The DNA was hydrolysed and the resultant nucleotides were analysed by reverse-phase HPLC [45].

#### DNA/DNA hybridization

DNA/DNA hybridization was carried out as described by De Ley *et al*. [46], with the modifications described by Huss *et al*. [47] and Escara & Hutton [48], using a model 2600 spectrophotometer equipped with a model 2527-R thermoprogrammer and plotter (Gilford Instrument Laboratories). Renaturation rates were compared with the TRANSFER.BAS program by Jahnke [49].

#### Ribotyping analysis

Standardized automated ribotyping is performed using the QualiconTM RiboPrinter system as described by Bruce [50]. The RiboPrinter system combines molecular processing steps for ribotyping in a stand-alone, automated instrument. Steps included cell lysis, digestion of chromosomal DNA with restriction enzyme *Eco*R1, separation of fragments by electrophoresis, transfer of DNA fragments to a nylon membrane, hybridization to a probe generated from the *rrn*B operon from *E. coli*, chemiluminescent detection of the probe to the fragments containing *rrn *operon sequences, image detection and computerized analysis of RiboPrint patterns.

#### Amplification of full-length thermostable lipase gene

Genomic DNA was extracted by using conventional method [51]. In order to amplify the full length sequence of thermostable lipase gene, a set of primers was designed based on the thermostable lipase gene sequence of *Bacillus thermoleovorans *(AF134840), as follows: BTL-F: 5'-GGC GGT GAT GGA ACG CTG CCA TGA-3' and BTL-R: 5'-CCG ACG ATA GAC TGG CGG ACA AAT G-3'. Polymerase Chain Reaction (PCR) was carried out in a reaction mixture (100 μl) containing DNA template (10–100 ng), 10 mM deoxynucleotide triphosphates (dNTPs) (0.2 mM), 10 × PCR buffer (10.0 μl), 25 mM MgCl_2 _(2 mM), oligonucleotide primers: BTL-F (30 pmol) and BTL-R (30 pmol), and *Taq *DNA polymerase (2 U). The gene was amplified with a thermocycler (Gene Amp PCR system 2400, Perkin Elmer, Foster, CA) with the temperature program of predenaturation at 94°C for 4 min, 30 cycles PCR of 1 min denaturation at 94°C, 2 min annealing at 55°C and 2 min extension at 72°C. The final extension step at 72°C was 7 min and preservation was at 4°C. The amplified products were electrophoresed on 1.0% agarose gel (w/v) with 1 kb marker as the standard marker. The PCR products were purified and sequenced as described earlier. The sequences alignment was generated using CLUSTALW and TEXSHADE in Biology Workbench 3.2 [52].

#### Protein pattern analysis

SDS-PAGE was done on 12% running gels by using the method of Laemmli [53]. A broad range of protein standard (MBI Fermentas, Germany) was used as a molecular mass marker. Extracellular protein was concentrated with 5,000 MWCO cut-off vivaspin 15R (Vivascience, Germany). Protein samples (30 μg) were loaded for analysis.

## Authors' contributions

TCL performed most of the experiments described in this paper, contributed to their design and analysis, and helped to draft the manuscript. RNZRAR, ABS and MB conceived of the study and experimental design, and prepared the manuscript. All authors read and approved the final manuscript.
